# The Acute Phase of Experimental Cardiogenic Shock Is Counteracted by Microcirculatory and Mitochondrial Adaptations

**DOI:** 10.1371/journal.pone.0105213

**Published:** 2014-09-04

**Authors:** Thor Allan Stenberg, Anders Benjamin Kildal, Espen Sanden, Ole-Jakob How, Martin Hagve, Kirsti Ytrehus, Terje S. Larsen, Truls Myrmel

**Affiliations:** 1 Cardiovascular Research Group, Department of Clinical Medicine, Faculty of Health Sciences, University of Tromsø, Tromsø, Norway; 2 Cardiovascular Research Group, Department of Medical Biology, Faculty of Health Sciences, University of Tromsø, Tromsø, Norway; 3 Department of Cardiothoracic and Vascular Surgery, University Hospital of North Norway, Tromsø, Norway; Georgia Regents University, United States of America

## Abstract

The mechanisms contributing to multiorgan dysfunction during cardiogenic shock are poorly understood. Our goal was to characterize the microcirculatory and mitochondrial responses following ≥10 hours of severe left ventricular failure and cardiogenic shock. We employed a closed-chest porcine model of cardiogenic shock induced by left coronary microembolization (n = 12) and a time-matched control group (n = 6). Hemodynamics and metabolism were measured hourly by intravascular pressure catheters, thermodilution, arterial and organ specific blood gases. Echocardiography and assessment of the sublingual microcirculation by sidestream darkfield imaging were performed at baseline, 2±1 and 13±3 (mean±SD) hours after coronary microembolization. Upon hemodynamic decompensation, cardiac, renal and hepatic mitochondria were isolated and evaluated by high-resolution respirometry. Low cardiac output, hypotension, oliguria and severe reductions in mixed-venous and hepatic O_2_ saturations were evident in cardiogenic shock. The sublingual total and perfused vessel densities were fully preserved throughout the experiments. Cardiac mitochondrial respiration was unaltered, whereas state 2, 3 and 4 respiration of renal and hepatic mitochondria were increased in cardiogenic shock. Mitochondrial viability (RCR; state 3/state 4) and efficiency (ADP/O ratio) were unaffected. Our study demonstrates that the microcirculation is preserved in a porcine model of untreated cardiogenic shock despite vital organ hypoperfusion. Renal and hepatic mitochondrial respiration is upregulated, possibly through demand-related adaptations, and the endogenous shock response is thus compensatory and protective, even after several hours of global hypoperfusion.

## Introduction

Cardiogenic shock (CS) is the result of a complex process with failure of O_2_ delivery, generalized ATP deficiency, and multiorgan dysfunction initiated by cardiac pump failure [1]. CS complicates 2–10% of acute coronary syndromes and is the leading cause of death among these patients, with a short-term mortality of 40–80% [2]. Several large trials have demonstrated that coronary revascularization is the most important strategy to improve patient survival [3]–[5]. However, patients who develop CS despite acute revascularization have a poor prognosis [6]–[8].

Interestingly, microcirculatory and mitochondrial dysfunction has been linked to the prognosis in septic shock [9], and several lines of research have led to the concept of a microcirculatory and mitochondrial dysfunction syndrome [10]–[12]. However, the support for such a syndrome in cardiogenic shock is more vague. Using venous plethysmography, Kirschenbaum et al. showed that forearm blood flow at rest and reactive hyperemia were diminished in a small subset of CS patients [13]. Reductions in the proportion of perfused small vessels and perfused capillary density have also been observed in patients with CS using orthogonal polarization spectral and sidestream darkfield imaging (SDF) imaging [14], [15]. However, mortality has also been observed in CS patients with unaltered microcirculation [15]. Finally, although cardiac mitochondria are impaired in left ventricular failure [16]–[18], extracardiac mitochondrial function has not been studied in pure pump-failure CS.

In this study, an experimental model without drug treatment was established to assess microcirculatory and mitochondrial responses to CS. We examined microcirculatory function, organ metabolism and mitochondrial respiration in a porcine model of acute left ventricular dysfunction and CS induced by coronary microembolization (CME). We show that sublingual microcirculatory function was well preserved during the acute phase following induction of shock and that a compensatory upregulation of renal and hepatic mitochondrial respiration occurred despite severe systemic hypoperfusion.

## Materials and Methods

The experimental protocol was approved by the local steering committee of the Norwegian Animal Research Authority and was conducted in accordance with the *Guide for the Care and Use of Laboratory Animals* published by the US National Institutes of Health (NIH Publication No. 85–23, revised 1996). 18 castrated male domestic pigs (Norwegian Landrace and Yorkshire hybrids) weighing 49±3 kg were adapted to the animal facilities for 4–7 days and fasted overnight before the experiment with free access to water.

### Anesthesia and instrumentation

The animals were premedicated with 20 mg/kg ketamine, 0.5 mg/kg midazolam and 0.03 mg/kg atropine administered intramuscularly. General anesthesia was induced with 0.01 mg/kg fentanyl and 10 mg/kg sodium pentobarbital through an ear vein and maintained with 0.02 mg/kg/h fentanyl, 4 mg/kg/h sodium pentobarbital and 0.3 mg/kg/h midazolam through a central venous catheter. The animals were intubated and mechanically ventilated using a volume-controlled ventilator adjusted according to capnography and arterial blood gases. The circulating volume was maintained by a 10 ml/kg/h saline infusion. 2500 IU heparin and 6 mg/kg amiodarone were administered to avoid catheter clotting and malignant arrhythmias. Arterial blood glucose was maintained at 4–6 mmol/l by infusion of 20% glucose as required. A normal core temperature was maintained at 38°C using a thermal mattress. A series of introducer sheaths were placed in the external jugular veins, femoral arteries and veins to facilitate vascular recordings and interventions as follows: I) A 7 Fr thermodilution catheter through the left external jugular vein to measure central venous pressure (CVP), mean pulmonary artery pressure (MPAP), pulmonary artery occlusion pressure (PAOP), cardiac index (CI), mixed venous O_2_ saturation (SvO_2_), and core temperature; II) Measurement of mean arterial pressure (MAP) and arterial blood sampling. III) Coronary artery catheterization, fluoroscopy and microembolization. IV) Catheters inserted via both femoral veins and advanced with fluoroscopic guidance into the hepatic vein and the left renal vein to obtain blood samples. A 10 Fr suprapubic catheter was used to drain the urinary bladder.

### Experimental protocol

The experiments were performed in an alternating manner between shock (n = 12) and time-matched controls (n = 6). Echocardiographic and microcirculatory recordings were obtained at baseline, 2 hours after CME, and ≥10 hours after CME, closely preceding circulatory collapse. The main trunk of the left coronary artery was catheterized with a 4 Fr angiography catheter under fluoroscopic guidance, and CME was performed using 50 µm polystyrene microspheres (Chromosphere, Duke Scientific Corporation) dissolved in 0.9% sodium chloride and 0.01% Tween 80. The degree of contractile dysfunction was titrated to a 30% decrease in CI through repeated injections of 10–15 mg every 5–10 min. The prespecified inclusion criteria for the CS group were: I) ≥30% acute reduction in CI; II) hypotension with MAP<65 mmHg or systolic pressure <90 mmHg; III) hypoperfusion as evidenced by oliguria/anuria; and IV) an observation time of ≥10 hours following CME. After the final measurements were performed, tissue samples were collected from the liver, the right kidney and the left ventricle, and the animals were euthanized with an intravenous injection of 20 mg/kg sodium pentobarbital.

### Speckle tracking echocardiography

Left ventricular short-axis transthoracic 2D images were acquired using a 5–12 MHz probe and a Philips iE33 ultrasound scanner (Philips Medical Systems). High frame rates (90–100 Hz) were achieved without a reduction in spatial resolution using a narrow image-sector angle and maximal zooming. Aortic flow was acquired by Doppler, and the timing of aortic valve opening and closure was determined to calculate strain in the ejection period. The 2D images were analyzed using dedicated offline software (Syngo Velocity Vector Imaging, Siemens Medical Systems). The software acquired and averaged three cardiac cycles. Short-axis 2D images were divided into 6 regions of interest with 8 tracked points per region of interest. Strain and strain rate values were determined at the time of aortic valve closure. Strain was defined by the Lagrangian formula ε = (L−L_0_)/L_0_ = ΔL/L_0_ where ε is strain, L_0_ is the initial length, and L is the instantaneous length. Furthermore, strain is expressed as the percentage of initial length such that positive values are observed with lengthening or thickening (i.e. radial thickening), and negative values are observed with shortening (i.e. longitudinal and circumferential shortening). Strain rate was defined as ε′ = Δ ε/Δt where ε′ is strain rate, and t is time [19]. All 2D images were assessed for quality prior to inclusion and image analysis, and the correct tracking of endo- and epicardial borders was controlled visually.

### Microcirculatory recordings and analysis

The sublingual microcirculation was evaluated using SDF imaging with a 5× objective lens (Microscan, Microvision Medical), and image analysis was conducted using a commercially available software package (Automated Vascular Analysis, Microvision Medical). At each measurement, steady images lasting ≥20 seconds were obtained from 5–10 sublingual areas, with care taken to avoid pressure artifacts by ensuring that venular flow was present throughout the recording [20]. SDF images were recorded electronically for offline analysis. All discernible vessels were manually demarcated in the microcirculatory video recordings according to current guidelines [20]. The total vessel density (TVD; mm/mm^2^) was calculated as the total length of vessels divided by the total surface area. Perfusion was qualitatively described as present (continuous flow for ≥20 seconds), absent (no flow for ≥20 seconds) or intermittent (no flow ≥50% of the time). Vessels with diameters less than 20 µm were defined as small vessels. Three equidistant horizontal and vertical lines were drawn on the recorded area, and the proportion of perfused small vessels (PPV) was calculated as the number of perfused small vessels (continuous flow ≥20 seconds) crossing the lines divided by the total number of small vessels crossing the lines. Perfused vessel density (PVD; mm/mm^2^), an estimate of functional capillary density, was calculated by multiplying the small vessel density by the proportion of perfused small vessels. The predominant type of small vessel flow (0 =  absent, 1 =  intermittent, 2 =  sluggish, and 3 =  normal) in each quadrant was assessed, and the microvascular flow index (MFI) was calculated as the averaged value from all four quadrants [21]. To obtain mean values at each measurement point, the data from 5 sublingual areas were averaged. The images used for these analyses were selected according to sharpness and the absence of movement artifacts to optimize image quality.

### Mitochondrial high-resolution respirometry

Cardiac subsarcolemmal (SSM) and interfibrillar mitochondria (IFM) were isolated from the left ventricle as previously described [22], [23]. Renal and hepatic mitochondria were also isolated by the procedure used to isolate SSM mitochondria. Mitochondrial respiration was measured using a Clark-type electrode (Oxygraph-2k, Oroboros Instruments). Complex 1-dependent respiration was assessed using glutamate and malate as substrates. Complex 2-dependent respiration was assessed using succinate as a substrate, and rotenone was added to inhibit complex 1 activity. The initial O_2_ consumption after the addition of mitochondria and substrate was recorded as state 2. State 3 respiration was obtained after the addition of ADP to a final concentration of 300 µM, and state 4 resting respiration was measured after the added ADP was depleted. The respiratory control ratio (RCR; state 3/state 4) was calculated as described by Chance et al. [24]. The ADP to oxygen (ADP/O) ratio was calculated based on the addition of a known amount of ADP and the associated O_2_ consumption. Mitochondrial protein concentration was determined by the Bradford method using bovine serum albumin as a standard [25]. Citrate synthase specific activity was calculated spectrophotometrically [26].

### Statistics

Hemodynamic and metabolic data are presented as mean±SD, whereas microcirculatory and mitochondrial data are presented as median±range. Hemodynamic, echocardiographic, metabolic and microcirculatory data were analyzed using a linear mixed-models approach with a restricted maximum likelihood method and the subject identifier as the random effect. Within-group trends that were not identified as significant using linear mixed-models were also assessed by one-way repeated measurements analysis of variance. P-values were adjusted for multiple comparisons using Tukey’s test. Mitochondrial respiratory data were assessed using the Mann-Whitney U-test. P-values <0.05 were regarded as statistically significant, and all analyses were conducted in JMP 9.0 (SAS Institute).

## Results

A total of 18 animals were used in this study. CME was performed in 12 animals, while 6 animals served as time-matched controls. Of the animals subjected to CME, 3 animals died shortly after CME due to fatal cardiac arrhythmia and abrupt circulatory collapse, 2 animals failed to develop low cardiac output, and 1 animal did not show evidence of hypoperfusion. Thus, 6 animals did not fulfill the inclusion criteria and were excluded. Acute measurements after CME were performed when the animals exhibited a hemodynamic steady state (2±1 hours), and the final in vivo measurements were performed 13±3 hours after CME. This time point closely preceded decompensation and cardiovascular collapse, and most animals in the shock group died during the interval of interest due to circulatory instability, escalating and ultimately fatal arrhythmias.

### Hemodynamics and metabolism

**Figure 1:**
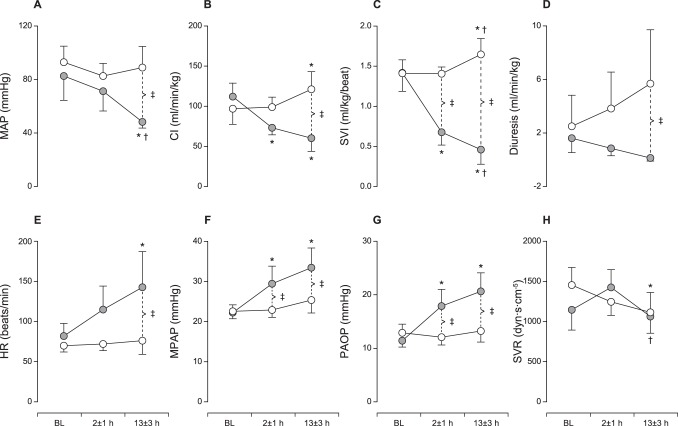
Hemodynamics. A–D: Mean arterial pressure (MAP), cardiac index (CI) and stroke volume index (SVI) decreased following coronary microembolization (CME). Diuresis gradually declined, and all shock-treated animals developed oliguria/anuria. E–F: A profound increase in heart rate (HR) developed after CME. Mean arterial pulmonary pressure (MPAP) and pulmonary artery occlusion pressure (PAOP) both increased after CME due to severe left ventricular dysfunction. The systemic vascular resistance (SVR) was unaffected by CME. The data are presented as mean±SD. Open circles, time-matched control group; filled circles, cardiogenic shock group. * p<0.05 within group compared to baseline, ^†^p<0.05 within group compared to 2±1 hours, ^‡^p<0.05 between groups.

**Figure 2:**
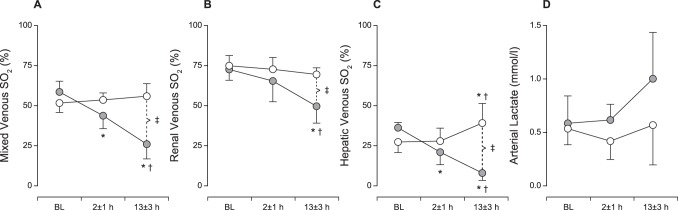
Metabolism. A–C: Mixed venous, renal venous and hepatic venous O_2_ saturations (SO_2_) were reduced following coronary microembolization. D: No increase in arterial lactate was observed despite profound shock. The data are presented as mean±SD. Open circles, time-matched control group; filled circles, cardiogenic shock group. * p<0.05 within group compared to baseline, ^†^p<0.05 within group compared to 2±1 hours, ^‡^p<0.05 between groups.

The groups were similar at baseline with respect to hemodynamic, metabolic and microcirculatory parameters. The microsphere dose used to induce left ventricular (LV) failure was 67±19 mg. As shown in Figure 1, the stroke volume index decreased to approximately 1/3 of the baseline value. LV failure was also evident by the increases in MPAP and PAOP. CVP increased in the shock group (7±1 vs. 12±2 mmHg, p<0.05). Despite compensatory tachycardia, an acute and sustained 40% reduction in CI was observed. Hypotension and oliguria/anuria developed gradually. The systemic vascular resistance (SVR) was similar between groups throughout the experiment. The systemic O_2_ delivery was reduced by approximately 50% in CS (6.5±1.7, ml/min/kg) compared with baseline and control group values (12.9±2.6 and 13.4±2.7, both p<0.05), whereas the systemic O_2_ consumption was unaltered. Figure 2 shows that both mixed and organ-specific venous O_2_ saturations declined substantially, with hepatic O_2_ saturation as low as 3%, indicating an extreme extraction across the liver, while no increase in arterial lactate concentration was observed. Similarly, there was no increase in renal and hepatic venous lactate levels (data not shown).

### Speckle tracking echocardiography

Short-axis transthoracic 2D images were excluded from speckle tracking analysis in 1 shock and 2 control animals due to technical reasons. The average LV strain and strain rate in the shock group tended to decrease in the radial dimension and increase in the circumferential dimension, implying impaired contractility (Figure S1). The anteroseptal and lateral regions of interest were most severely affected by CME, and there was no evidence of compensatory hyperkinesia in regions supplied by the right coronary artery (Video S1).

### Microcirculatory function

**Figure 3:**
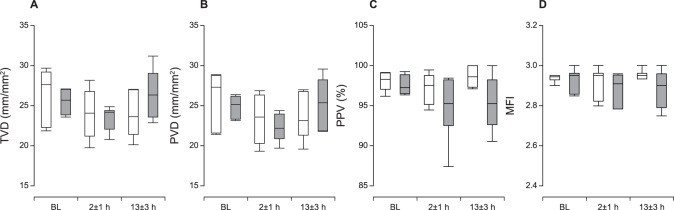
Total and Perfused Small Vessel Densities. A–B: The total small vessel density (TVD) and perfused small vessel density (PVD) were fully preserved after coronary microembolization (CME). C: The proportion of perfused small vessels (PPV) was also identical across time points and between groups. D: The microvascular flow index (MFI) was not affected by CME. The data are presented as median±range. Open box plots, time-matched control group; filled box plots, cardiogenic shock group.

The sublingual total large vessel density, small vessel density, proportion of perfused small vessels and microvascular flow index did not change throughout the experiment in either group, and the shock group exhibited no detectable defects in microcirculatory function as assessed by SDF (Figure 3). Recordings from a pilot experiment are presented in Video S2, demonstrating no difference in microcirculatory parameters during shock. Furthermore, the effect of administering high-dose arginine-vasopressin on the microcirculation is demonstrated in the video to illustrate the sensitivity of SDF imaging in detecting potential alterations in microcirculatory function.

### Mitochondrial respiration

LV, renal and hepatic mitochondria were isolated from all animals. Measurements of complex 2-dependent respiration in SSM from 1 control animal and 1 shock animal were excluded from analysis due to technical issues, thus n = 5 for these measurements. The respiratory rates were normalized to citrate synthase activity in the samples to permit the comparison of measurements from different suspensions of isolated mitochondria. All measurements were also compared after adjustment for the total protein content, with similar results.

Complex 1-dependent respiration in the cardiac IFM and SSM fractions were unaffected by CME, whereas complex 1-dependent renal and hepatic state 2, 3 and 4 respiratory rates were significantly elevated following CME (Table 1). The complex 2-dependent respiratory rates in the cardiac IFM and SSM fractions were also similar between groups. Complex 2-dependent hepatic state 2 and renal state 3 and 4 respiratory rates were significantly elevated in the shock group. Mitochondrial coupling and viability, as assessed by the RCR, were similar between groups. The ADP/O ratio was also unaffected by CME, indicating a similar mitochondrial efficiency and thus no difference in non-metabolic O_2_ consumption between groups.

**Table 1:** Mitochondrial Respiratory Parameters.

			Organ			
Substrate		Group	Cardiac IFM	Cardiac SSM	Renal	Hepatic
Glutamate						
	State 2	Control	59 (39–65)	38 (22–44)	90 (78–104)	77 (53–86)
		Shock	50 (14–80)	27 (15–36)	116 (105–132)‡	94 (79–142)‡
	State 3	Control	434 (241–473)	393 (246–700)	955 (678–1138)	947 (391–1193)
		Shock	311 (73–702)	222 (101–517)	1371 (1103–1885)‡	1275 (1124–1987)‡
	State 4	Control	88 (62–104)	56 (39–84)	89 (76–108)	59 (53–104)
		Shock	105 (68–180)	62 (55–62)	110 (105–149)‡	116 (102–160)‡
	RCR	Control	4.6 (3.9–7.6)	6.8 (4.9–9.7)	10.4 (8.4–11.5)	10.4 (7.5–12.5)
		Shock	2.7 (1.1–6.3)	3.7 (1.6–8.8)	12.5 (10.1–13.1)	12.0 (9.4–12.5)
	ADP/O	Control	2.9 (2.7–3.1)	2.8 (2.7–3.0)	2.6 (2.4–2.9)	2.7 (2.5–3.1)
		Shock	2.0 (1.5–3.0)	2.5 (2.1–3.0)	2.6 (2.4–2.9)	2.8 (2.5–2.9)
Succinate						
	State 2	Control	165 (103–221)	131 (92–283)	353 (304–416)	208 (161–228)
		Shock	154 (50–269)	82 (39–128)	415 (379–474)	249 (198–352)‡
	State 3	Control	345 (210–406)	336 (190–541)	1752 (1560–1953)	1531 (1209–1862)
		Shock	267 (91–550)	199 (99–338)	2440 (2018–2516)‡	1892 (1344–2614)
	State 4	Control	148 (114–254)	148 (98–329)	212 (137–224)	232 (156–255)
		Shock	168 (85–297)	113 (83–128)	253 (189–335)‡	275 (225–394)
	RCR	Control	1.8 (1.6–2.3)	2.2 (1.6–3.0)	8.7 (7.5–12.5)	7.3 (6.0–8.0)
		Shock	1.5 (1.1–2.1)	2.0 (1.2–2.7)	9.1 (7.3–11.0)	6.5 (5.8–7.9)
	ADP/O	Control	1.7 (1.6–2.1)	1.8 (1.4–2.1)	1.8 (1.7–2.0)	1.9 (1.6–2.0)
		Shock	1.2 (0.1–1.8)	1.7 (0.4–1.9)	1.9 (1.8–2.0)	1.9 (1.8–2.0)

Renal and hepatic complex 1-dependent state 2, 3 and 4 mitochondrial respiration using glutamate as substrate were increased in cardiogenic shock, whereas respiratory control ratio (RCR) and ADP/O values were unaffected indicating well-coupled and efficient respiration. Cardiac interfibrillar (IFM) and subsarcolemmal (SSM) mitochondrial respiration was unaffected. Complex 2-dependent mitochondrial respiration using succinate as substrate demonstrates a similar tendency. State 2, 3 and 4 respiratory rates are presented as pmol O_2_/min/IU citrate synthase. The data are presented as median±range.

‡ p<0.05 between groups.

### Biochemistry

**Figure 4:**
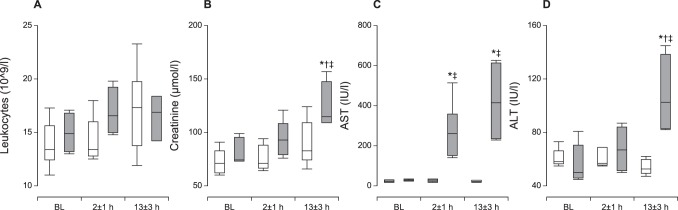
Indicators of Organ Damage. A: Circulating leukocyte levels were similar between groups, indicating the absence of any major inflammatory response after coronary microembolization (CME). B: Creatinine levels increased following CME, in parallel with the kidney failure evidenced by oliguria/anuria. C–D: Hepatic aspartate (AST) and alanine (ALT) transaminase levels increased after CME, indicating hepatocellular damage. The data are presented as median±range. Open box plots, time-matched control group; filled box plots, cardiogenic shock group. * p<0.05 within group compared to baseline, ^†^p<0.05 within group compared to 2±1 hours,^ ‡^p<0.05 between groups.

The level of circulating leukocytes was similar in both groups across all time points, indicating the absence of any major systemic inflammatory response. Creatinine levels were increased in the shock group, corresponding to the development of oliguria and kidney failure. Aspartate and alanine transaminase levels both increased in the shock group, with the latter indicating hepatocellular damage (Figure 4). Cardiac troponin T levels increased to 5.8±2.8 µg/l 13±3 hours after CME, whereas they remained below the detection limit (0.01 µg/l) in the control group.

## Discussion

In these healthy young animals, the reduced systemic O_2_ delivery in the acute phase of experimental CS is counteracted by microcirculatory and mitochondrial adaptations and this peripheral adaptability is sufficient to withstand anaerobic metabolism despite prolonged severe hypoperfusion. CME induced severe decreases in stroke volume index, low CI, hypotension and a gradual onset of oliguria/anuria. This untreated CS was ultimately fatal. Despite acute and prolonged hypotension, the SVR was unaltered in the shock group. This has been observed by others both experimentally and clinically and challenges the classical paradigm of CS [27]–[29]. We found no biochemical indication of an acute inflammatory response as a potential counteraction of the classical sympaticoneural vasoconstriction, but a systemic inflammatory response could still contribute to the lack of a vasoconstrictive response.

Microvascular dysfunction has been implicated in the pathophysiology of CS in small-sample studies of CS patients [13], [14]. In a study of 68 patients with CS following acute myocardial infarction, below median sublingual perfused capillary densities were associated with the development of multiorgan failure and poor outcomes [15]. From a pathophysiological point of view, it is difficult to appreciate the significance of this observation. The study population was heterogeneous, and the patients were treated with inotropes, vasoactive drugs and mechanical assist systems. Thus, the microvascular impairment in these patients may have been an epiphenomenon associated with the treatment modalities, which is of limited relevance to the underlying pathophysiological cascades. In support of this theory, small vessel flow has been shown to improve upon the withdrawal of intraaortic balloon counterpulsation in patients weaned from mechanical support [30]. Also, vasopressor addition profoundly affected the microcirculatory function in our experimental model, as shown by Video S2. We observed no detectable microcirculatory dysfunction in untreated and severe CS, and the sublingual total large vessel density, small vessel density, proportion of perfused small vessels and microvascular flow index were fully preserved throughout the experiment. Interestingly, these findings could be related to the observed upregulation of mitochondrial function, as the metabolic demand is maintained. During sepsis, diminished tissue O_2_ consumption and a parallel increase in tissue O_2_ levels are caused by mitochondrial dysfunction [31], in turn reducing the levels of vasodilating metabolites and thus possibly contributing to decreased microvascular flow. Similar alterations with reduced microvascular flow and perfused capillary density are observed in normobaric hyperoxia, indicating that O_2_ availability may control microvascular perfusion [32]. Thus, our data, which demonstrate the full maintenance of microcirculatory function during CS, can be explained by tissue hypoxia concurrent with unimpeded mitochondrial metabolism and thus high O_2_ demand.

The effect of CS on extracardiac mitochondrial function has received limited attention. CS is characterized by several detrimental processes, with inflammation, hypoxia and ischemia-reperfusion all contributing to systemic cellular injury [33]. As stated, in the present study, however, we observed no indication of any major systemic inflammatory response. In contrast, assessment of the O_2_ saturation in the parenchymal venous drainage revealed organ hypoxia to such an extent that enzyme leaks were present. Thus, the effects of hypoxia could parallel organ pathology in CS. Notably, state 3 respiratory rate in cardiac mitochondria has been found to increase more than 100% during acute in vivo hypoxemia and to stabilize at more than 40% above baseline during chronic in vivo hypoxemia [34]. A similar observation has been made following hypoperfusion [35], [36], and comparable effects were also observed when demand was increased by endurance training [37], [38]. Hypoxia activates hypoxia-inducible factor 1 (HIF-1) through increased generation of reactive oxygen species (ROS). HIF-1 initiates a potentially adaptive response where increased glycolytic flux and pyruvate-lactate conversion, impaired tricarboxylic-acid-cycle activity and altered composition of cytochrome c oxidase (COX, complex 4) in the electron transport chain ensures an efficient ATP production without excessive ROS generation [39]. Noticeably, through subunit conversion from COX4-1 to COX4-2, O_2_ consumption and ATP production increases under hypoxic conditions, whereas H_2_O_2_ levels decrease [40]. These changes in mitochondrial oxidative phosphorylation capacity appear to be adaptive and imply that mitochondrial efficiency is increased under hypoxic conditions. Similar mechanisms may increase ATP production due to increased demand following acute exposure to inflammatory cytokines [41]. However, in vitro exposure of isolated mitochondria to hypoxic conditions produces variable effects on respiratory activity [42], [43], and it is likely that these demand-related adaptations are caused, at least in part, by extramitochondrial interactions [44], [45].

We observed an increase in renal and hepatic state 3 respiratory rates in CS using complex 1-dependent substrates, whereas this parameter was unaltered in both cardiac fractions. Renal and hepatic state 2 and 4 respiratory rates were slightly increased in the shock group, but the RCR values and ADP/O ratios were similar, indicating well-coupled and efficient respiration. A similar trend was observed when succinate was used as a complex 2-dependent substrate, but only renal state 3 respiratory rate was significantly elevated in the shock group. Cardiac respiratory data were highly variable, and this likely reflects the heterogeneous pattern of microinfarctions after CME [46]. Prior studies have demonstrated decreased respiratory rates in both the ischemic and remote myocardium [17], [18], and this has been linked to reduced levels of the supercomplex 1/3-dimer/4, together with increased phosphorylation of complex 4 subunits [18], [47]. Cardiac mitochondrial dysfunction and energetic failure are thus potentially important mechanisms propagating contractile failure during cardiogenic shock [16], [48], [49].

This study has several limitations when exploring potential mechanisms of human cardiogenic shock. The porcine model used young animals without comorbid disease and with substantial compensatory reserves. The unresuscitated observation period following the induction of shock is of limited duration, and it is possible that a longer observation phase, or an active treatment protocol, could have uncovered both microcirculatory and mitochondrial dysfunction. The methods used to isolate mitochondria from various tissues were designed to exclude damaged mitochondria and preserve the viable mitochondria. Thus, existing mitochondrial dysfunction in vivo may remain undetected. However, we observed no differences in total protein content or citrate synthase activity among groups, indicating that the amounts of mitochondria isolated from each group were similar. In cardiac biopsies, however, the loss of damaged mitochondria due to the isolation procedure likely contributed to the observed results. Furthermore, only two substrate combinations were used in the experiments. Glutamate and malate were used to observe complex 1-dependent respiration, and succinate was used as a substrate for complex 2-dependent respiration. Oligomycin was not used to inhibit ATP-synthase, but a stable state 4 was observed following ADP depletion. Thus, the use of other substrates may reveal differences between CS and controls that remained undetected in the present study.

In conclusion, we demonstrate that the microcirculation is preserved in a porcine model with untreated CS despite vital organ hypoperfusion. Renal and hepatic mitochondrial respiration is upregulated, possibly through demand-related adaptations, and the endogenous shock response appears to be compensatory and protective, even after several hours of global hypoperfusion.

## Supporting Information

Figure S1 **Strain and Strain Rate.** A–B: The average radial strain across all regions of interest decreased after coronary microembolization (CME), whereas a similar left ventricular dysfunction was evident by the increase in the average circumferential strain. C–D: The average circumferential strain rate also increased after CME. Data are presented as median±range. Open box plots, time-matched control group; filled box plots, cardiogenic shock group. *p<0.05 within group compared to baseline by one-way repeated measurements analysis of variance.
(EPS)

Video S1 **Example echocardiography recordings.** Short-axis transthoracic 2D images from a cardiogenic shock animal at baseline, after coronary microembolization and during cardiogenic shock.
(M4V)

Video S2 **Example microcirculatory recordings.** Sidestream darkfield images from a pilot experiment at baseline, during cardiogenic shock and after the administration of high-dose arginine-vasopressin.
(M4V)
